# Theoretical analysis on thermodynamic stability of chignolin

**DOI:** 10.1038/s41598-019-41518-1

**Published:** 2019-03-26

**Authors:** Tomonari Sumi, Kenichiro Koga

**Affiliations:** 1 Research Institute for Interdisciplinary Science, Okayama University, 3-1-1 Tsushima-Naka, Kita-ku, Okayama, 700-8530 Japan; 2 Department of Chemistry, Faculty of Science, Okayama University, 3-1-1 Tsushima-Naka, Kita-ku, Okayama, 700-8530 Japan

## Abstract

Understanding the dominant factor in thermodynamic stability of proteins remains an open challenge. Kauzmann’s hydrophobic interaction hypothesis, which considers hydrophobic interactions between nonpolar groups as the dominant factor, has been widely accepted for about sixty years and attracted many scientists. The hypothesis, however, has not been verified or disproved because it is difficult, both theoretically and experimentally, to quantify the solvent effects on the free energy change in protein folding. Here, we developed a computational method for extracting the dominant factor behind thermodynamic stability of proteins and applied it to a small, designed protein, chignolin. The resulting free energy profile quantitatively agreed with the molecular dynamics simulations. Decomposition of the free energy profile indicated that intramolecular interactions predominantly stabilized collapsed conformations, whereas solvent-induced interactions, including hydrophobic ones, destabilized them. These results obtained for chignolin were consistent with the site-directed mutagenesis and calorimetry experiments for globular proteins with hydrophobic interior cores.

## Introduction

Understanding the dominant factor behind thermodynamic stability of proteins remains a challenging issue in biochemistry, biophysics, and molecular biology^1–3^. Several theories explaining protein stability have been proposed. In 1936, Pauling and Mirsky suggested that a protein achieved a uniquely defined conformation held in place by N-H···O hydrogen bonds between the nitrogen and oxygen atoms in the peptide chain, the interaction energy of each bond being approximately 5 kcal/mol^4^. Three years later, Bernal suggested that the hydrophilic residues of a protein were exposed to the aqueous solution, whereas the hydrophobic parts were in contact with each other in the interior of the protein^5^. In 1951, Pauling’s group discovered the most important structural elements in globular proteins: alpha helices^6^ and beta sheets^7^. They, furthermore, pointed out that the backbone N-H and O forming intramolecular hydrogen bonds were approximately 2 kcal/mol more stable than those forming intermolecular hydrogen bonds with surrounding water molecules^7^. In 1959, Kauzmann concluded in his seminal review^8^ that hydrophobic attraction was a dominant factor in the thermodynamic stability of the folded conformation for many globular proteins. This has been supported by the following experimental observations: (i) the change in Gibbs energy for transferring a small nonpolar molecule from an aqueous solution to an organic solvent is large and negative^8^; (ii) the net effect of electrostatic interactions on protein stability is negligibly small^9^; and (iii) numerous nonpolar residues are indeed located in the interior of globular proteins^10,11^.

In the late 1980s, it became possible to examine the dominant factor behind protein stability by applying site-directed mutagenesis. It was shown that (1) both hydrophobic interactions^12^ and intramolecular hydrogen bonding^13^ contributed substantially to protein stability; (2) the enhancement of van der Waals interactions due to tight packing in the protein interior caused by the replacement of small hydrophobic residues with larger ones resulted in increased protein stability^14^; and (3) the effect of hydrogen bonding of peptide groups on protein stability was comparable to that of hydrogen bonding of side chains^13,15^. These observations suggest the importance of intramolecular interactions in protein stability. Nevertheless, the thermodynamic stability of proteins has been basically modeled according to Kauzmann’s hydrophobic interaction hypothesis (e.g., refs ^16–18^).

**Figure 1:**
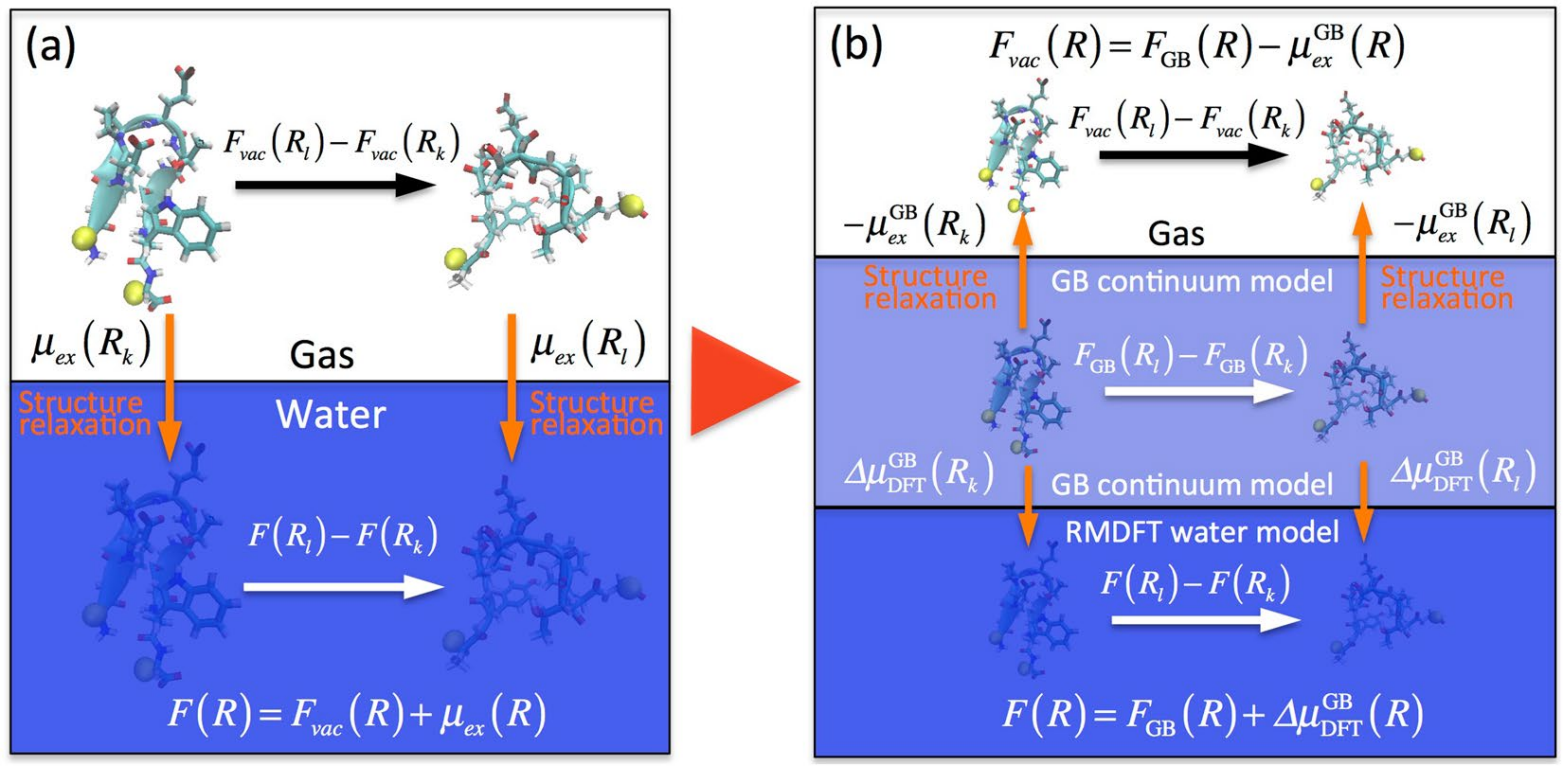
Calculation scheme of free energy profile of a protein. The distance between alpha carbon atoms at the C-terminus and N-terminus (shown as yellow spheres) is introduced as the coordinate *R* specifying the dimensions of the protein. (**a**) Thermodynamic cycle depicting the relationship in Eq. 1 among the free energy profile of the protein in water, *F*(*R*), the free energy profile in vacuum, *F*_*vac*_(*R*), and the excess chemical potential profile, *μ*_*ex*_(*R*), at a distance *R*. (**b**) Two different thermodynamic cycles, which allow for the calculation of *F*(*R*) and *F*_*vac*_(*R*), if the following are obtained: free energy profile determined by the generalized Born (GB) model umbrella sampling MD simulations with a dielectric constant *ε*_*r*_ = 80, *F*_GB_(*R*), excess chemical potential profile of the protein in the GB model with *ε*_*r*_ = 80 (Eq. 4), ${\mu }_{ex}^{{\rm{GB}}}(R)$, and free energy difference between the protein in water described by the GB model and that described by the RMDFT model (Eq. 7), ${\rm{\Delta }}{\mu }_{{\rm{DFT}}}^{{\rm{GB}}}(R)$.

Molecular dynamics (MD) simulations are a powerful tool that enables us to investigate large conformational changes in proteins^19–27^. Generalized ensemble MD simulations have been applied to calculate temperature and pressure dependence of free energy profile of proteins and peptides^24,25^. Long equilibrium MD simulations have been performed to characterize folding pathways and free energy changes^26,27^. In general, a free energy profile of a protein can be expressed as a function of a coordinate *R*:

1 $$F(R)={F}_{vac}(R)+{\mu }_{ex}(R),$$

where *F*_*vac*_(*R*) is the free energy profile in vacuum and *μ*_*ex*_(*R*) is the excess chemical potential profile of the protein, i.e., the free energy change for hydration, at the coordinate *R* (Fig. 1a). *F*_*vac*_(*R*) consists of intramolecular energy and entropy:

2 $${F}_{vac}(R)={E}_{vac}^{{intra}}(R)-T{S}_{vac}^{{intra}}(R).$$

*μ*_*ex*_(*R*) in Eq. 1, which acts as a solvent-induced interaction on the protein, can be expressed as

3 $${\mu }_{ex}(R)={\mu }_{nonpol}(R)+{\mu }_{pol}(R),$$

where *μ*_*nonpol*_(*R*) is the nonpolar part of *μ*_*ex*_(*R*), which is calculated by omitting all partial charges on the protein and *μ*_*pol*_(*R*) is the remaining polar part of *μ*_*ex*_(*R*). *μ*_*nonpol*_(*R*) is expected to provide the upper limit of the contribution of the solvent-induced hydrophobic interaction to the hydrophobic collapse of the protein since *μ*_*nonpol*_(*R*) includes the nonpolar contributions to *μ*_*ex*_(*R*) arising from all polar residues as well. Such a decomposition of *F*(*R*) should provide insights into understanding the dominant factor in the thermodynamic stability of the protein. Here, it can be seen that *μ*_*ex*_(*R*) is essentially different from the simple ensemble-average value of solvation free energy calculated using conformations generated under the coordinate *R* in the solvent. This is because *μ*_*ex*_(*R*) includes the effect of conformation relaxation during the gradual annihilation of either the protein or all the solvent molecules^28^ (see Fig. 1a). Although the importance of the conformation entropy in *F*_*vac*_(*R*) has widely been realized, the effect of conformation relaxation on *μ*_*ex*_(*R*) has been overlooked or simply ignored (see an example in the Supplementary Information); however, we have explicitly evaluated this as described below. Furthermore, through liquid-state DFT, it was easy to determine *μ*_*ex*_(*R*) and *μ*_*nonpol*_(*R*) (or *μ*_*pol*_(*R*)) as well as the intramolecular conformation entropy $T{S}_{vac}^{{intra}}(R)$, which would have been hard if we had employed explicit-solvent all-atom MD simulations.

In this study, we present an efficient computational method to evaluate the free energy profile and the components as a function of a coordinate *R* using a combination of continuum solvent MD simulations and a recently developed reference-modified density functional theory (RMDFT) for calculation of solvation free energy^29–32^. The reliability of RMDFT in calculating the solvation free energy has been demonstrated by comparing experiments on organic solute molecules^29,32^. In contrast to continuum solvent models, the present method allows for hydration effects to be taken into consideration at a molecular level. We applied this method to a small, designed protein, chignolin, consisting of ten amino acids with the sequence GYDPETGTWG^33^, and revealed a dominant factor responsible for the thermodynamic stability as well as temperature- and pressure-induced unfolding of chignolin.

## Computational Method

We calculated the free energy profile *F*(*R*) and its components *F*_*vac*_(*R*) and *μ*_*ex*_(*R*) for chignolin as a function of a coordinate *R*. The distance between the alpha carbon atoms at the C-terminus and the N-terminus was chosen as the coordinate *R*. It is not easy to determine the most suitable single reaction coordinate to characterize folding kinetics. However, we aimed to obtain the free energy profile *F*(*R*) as a function of the measure of the dimensions of chignolin, and we chose the end-to-end distance as *R* as it is one of the common measures of the dimensions of polymers. It should be noted that the free energy *difference* and its decomposition between folded and extended conformations do not depend on the pathway and depend only on the initial and final state. Thus, the conclusion based on the decomposed free energy differences is not affected by the choice of the coordinate parameter.

There are steep energy barriers associated with structural changes involving cleavage of intramolecular hydrogen bonding, which make it difficult to directly calculate the free energy profile in vacuum, *F*_*vac*_(*R*), using umbrella sampling. In fact, exchange of the intramolecular hydrogen bonding in vacuum is very rarer than in water due to the higher energy barriers since no water molecules are surrounding chignolin. We thus chose an alternative approach: first, we calculated the free energy profile *F*_GB_(*R*) in a continuum solvent with a dielectric constant of water (*ε*_*r*_ = 80), *F*_GB_(*R*), using implicit-solvent generalized Born (GB) umbrella sampling MD simulations (Fig. 1b), which are much faster than explicit-solvent all-atom umbrella sampling MD simulations; second, as shown later, we obtained *F*_*vac*_(*R*) from *F*_GB_(*R*).

The excess chemical potential profile for the GB model, ${\mu }_{{\rm{ex}}}^{{\rm{GB}}}(R)$, was calculated by the free energy perturbation method as follows:

4 $${\mu }_{{\rm{ex}}}^{{\rm{GB}}}(R)={\sum }_{a=0}^{n}[{\rm{\Delta }}{\mu }_{{\varepsilon }_{a+1}}^{{\varepsilon }_{a}}(R)-{\rm{\Delta }}{\mu }_{{\varepsilon }_{a}}^{{\varepsilon }_{a+1}}(R)]/2,$$

with

5 $${\rm{\Delta }}{\mu }_{{\varepsilon }_{j}}^{{\varepsilon }_{i}}(R)=-\,{k}_{B}T\,\mathrm{ln}\,{\langle \exp [-({\rm{\Delta }}{G}_{solv}^{{\rm{GB}}}({\varepsilon }_{j})-{\rm{\Delta }}{G}_{solv}^{{\rm{GB}}}({\varepsilon }_{i}))/{k}_{B}T]\rangle }_{R}^{{\varepsilon }_{i}},$$

where *n* is the number of intermediate states between *ε*_*r*_ = 1 (vacuum) and 80 (liquid water), ${\langle \cdots \rangle }_{R}^{{\varepsilon }_{i}}$ represents the ensemble average of conformations generated by GB MD simulations with a dielectric constant of *ε*_*i*_, in which the coordinate is fixed at *R*, and ${\rm{\Delta }}{G}_{solv}^{{\rm{GB}}}({\varepsilon }_{i})$ is the solvation free energy of chignolin calculated from the dielectric constant *ε*_*r*_ = *ε*_*i*_. It should be noted that the solvation free energy given for each conformation serves as a potential energy term in the effective Hamiltonian of GB MD simulations. Thus, the solvation free energy appears in Eq. 5 instead of the protein-water interaction energy that usually appears in explicit-solvent all-atom MD free energy perturbation calculations (Appendix A in the Supplementary Information). As shown in Fig. 1b, the free energy profile in vacuum, *F*_*vac*_(*R*), can be obtained from

6 $${F}_{vac}(R)={F}_{{\rm{GB}}}(R)-{\mu }_{{ex}}^{{\rm{GB}}}(R).$$

An interval of *ΔR* = 0.1 nm was employed, and thus, steep changes in *F*_*vac*_(*R*), included within an interval less than *ΔR*, were fully or partially omitted. However, this was not problematic because we focused on the overall profile of *F*_*vac*_(*R*).

The GB model is computationally favorable, but it is less accurate and tends to underestimate solvation of polar groups^34–36^. To obtain a more reliable free energy profile, we calculated the free energy difference between the solute in water described by the GB model and that described by the RMDFT model,

7 $${\rm{\Delta }}{\mu }_{{\rm{DFT}}}^{{\rm{GB}}}(R)=-\,{k}_{B}T\,\mathrm{ln}\,{\langle \exp [-({\rm{\Delta }}{G}_{hyd}^{{\rm{DFT}}}-{\rm{\Delta }}{G}_{solv}^{{\rm{GB}}}({\varepsilon }_{r}=80))/{k}_{B}T]\rangle }_{R}^{{\varepsilon }_{r}=80},$$

where ${\rm{\Delta }}{G}_{hyd}^{{\rm{DFT}}}$ is the hydration free energy according to the RMDFT model and includes the effects of temperature and pressure^32^. Finally, the free energy profile in water, *F*(*R*), and the excess chemical potential profile, *μ*_*ex*_(*R*), were obtained from

8 $$F(R)={F}_{{\rm{GB}}}(R)+{\rm{\Delta }}{\mu }_{{\rm{DFT}}}^{{\rm{GB}}}(R)$$

and

9 $${\mu }_{ex}(R)={\mu }_{{ex}}^{{\rm{GB}}}(R)+{\rm{\Delta }}{\mu }_{{\rm{DFT}}}^{{\rm{GB}}}(R),$$

respectively (Fig. 1b). It is noted that we can use the free energy perturbation by Eq. 7 to calculate *F*(*R*) at high pressures, if the ensemble average ${\langle \cdots \rangle }_{R}^{{\varepsilon }_{r}=80}$ includes enough conformations that becomes important at the high pressures. The validity is all the free energy perturbation calculations used above should be assessed by the standard error. We were now able to evaluate separately the two components of the free energy profile, namely the purely intramolecular part, *F*_*vac*_(*R*), and the solvent-induced part, *μ*_*ex*_(*R*). These were further decomposed as discussed below.

## Results

### Decomposition of the free energy profile

**Figure 2:**
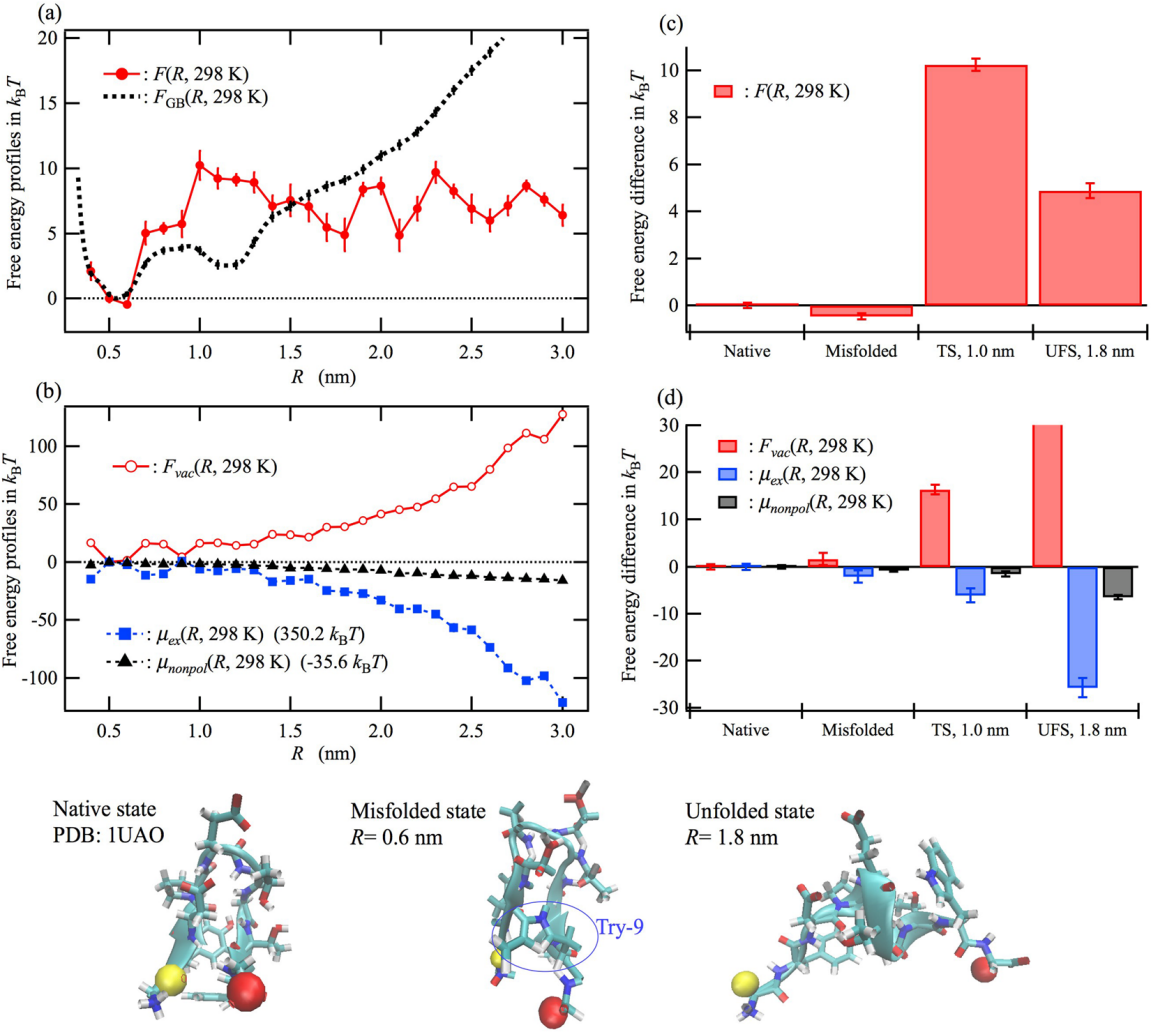
Free energy profiles of chignolin in water at 298 K. All profiles are shown in *k*_B_*T* and are shifted vertically, so that the value becomes zero at a distance of *R* = 0.5 nm, which corresponds to the native state. (**a**) Free energy profile calculated according to the GB model, *F*_GB_(*R*), and that corrected by the RMDFT method, *F*(*R*). (**b**) Free energy profile in vacuum, *F*_*vac*_(*R*), excess chemical potential profile, *μ*_*ex*_(*R*), and nonpolar part of *μ*_*ex*_(*R*), *μ*_*nonpol*_(*R*), calculated by removing all partial charges on chignolin (see Computational Details). (**c**,**d**) These free energy differences from the native state (*R* = 0.5 nm) are shown for the misfolded state at *R* = 0.6 nm, the transition state (TS) at *R* = 1.0 nm, and an unfolded state (UFS) at *R* = 1.8 nm. The numbers beside the legends in (**b**) indicate the vertically shifted value for these profiles. Here and hereafter, the error bars for all profiles indicate the standard error. The shown ternary structures are for the native state from the Protein Data Bank database (PDB: 1UAO), misfolded state obtained at *R* = 0.6 nm, and unfolded state obtained at *R* = 1.8 nm. The yellow and red spheres depict the alpha carbon atoms at the C-terminus and N-terminus, respectively. In the misfolded state, the relative position of Try-9 compared with Try-2 is different from that of the native state because of rotation in backbone torsion angle *ψ* for Gly-7^39^.

The curves in Fig. 2a represent the free energy profiles *F*(*R*) and *F*_GB_(*R*) at 298 K. The distance *R* = 0.5 nm corresponds to the native state, and these free energy profiles are plotted, so that the value becomes zero at this distance. The misfolded state^37–41^ is found at distances around *R* = 0.6 nm (see Fig. S1 in the Supplementary Information) due to inaccurate force field parameters for glycine backbone^39^. The free energy profile corrected by the RMDFT method, *F*(*R*), sharply increases and then reaches a plateau as the distance *R* increases from 0.5 nm, whereas *F*_GB_(*R*) gradually increases. There are some small minima in the plateau region of *F*(*R*) (e.g., *R* = 1.8, 2.1, and 2.6 nm). A similar plateau in *F*(*R*) has been reported by a generalized-ensemble all-atom MD simulation with explicit solvent by Okumura^25^. The free energy difference between the native and denatured states was determined by Okumura to be 4.6 *k*_B_*T*. This value is quantitatively consistent with the 4.9 *k*_B_*T* observed at the minimum on the plateau region of *F*(*R*). This quantitative agreement shows the validity of the present method, at least, for chignolin. In contrast, the free energy profile of the GB model, *F*_GB_(*R*), increased with increasing *R* for *R* > 1.3 nm. This substantial difference indicates the necessity of using the RMDFT method to describe the solvation of chignolin in water.

The curves in Fig. 2b show the free energy profile in vacuum, *F*_*vac*_(*R*), the excess chemical potential profile, *μ*_*ex*_(*R*), and the nonpolar part of *μ*_*ex*_(*R*), *μ*_*nonpol*_(*R*), at 298 K. *μ*_*ex*_(*R*) decreases with increasing *R*, whereas *F*_*vac*_(*R*) increases. Thus, *μ*_*ex*_(*R*), namely, the solvent-induced part of *F*(*R*), appears to stabilize the unfolded state. Indeed, earlier theoretical studies on small peptides^42,43^ and several proteins^44–46^ predicted a lower solvation free energy for unfolded conformations than for folded ones. In addition, the nonpolar part *μ*_*nonpol*_(*R*) also decreases with increasing *R*, indicating that the solvent-induced hydrophobic interaction *μ*_*nonpol*_(*R*), which gives the upper limit of the hydrophobic contribution of *μ*_*ex*_(*R*), also stabilizes the unfolded state rather than the folded state. This result qualitatively agrees with MD simulations of peptides by Kokubo *et al*.^43^ A similar solvent-induced repulsive force has also been shown for large hydrophobic molecules (e.g., fullerene C_60_) in water^44,45^. The obtained results for chignolin indicate that the *intramolecular* interactions including van der Waals and electrostatic forces are a dominant factor in the collapse of chignolin because the intramolecular-force-driven collapse slightly conquers the solvent-induced expansion. This observation is consistent with large-scale molecular dynamics simulations for multi-peptide aggregations that are predominantly caused by both van del Waals and Coulomb interactions between hydrophobic amino acids^46^. Furthermore, it is implied that a subtle balance between the competitive factors, i.e., the intramolecular interactions and the solvent-induced interactions determines the conformation of the native state. These results are inconsistent with Ben-Naim’s theory where hydrophilic interactions play more important role on protein stability rather than hydrophobic ones^2,47,48^; however, we agree with him in terms of the importance of electrostatic interactions in protein stability.

Figure 2c,d plot the free energy differences from the native state (*R* = 0.5 nm) for the misfolded state (*R* = 0.6 nm), transition state (*R* = 1.0 nm), and unfolded state (*R* = 1.8 nm). *F*_*vac*_(*R*) and *μ*_*ex*_(*R*) are respectively increased and decreased at the misfolded state, implying that the relative stability between the native and misfolded state is determined as a result of the competition between the intramolecular interactions and the solvent-induced interactions. From the comparison with the unfolded state at *R* = 1.8 nm, the elevation of *F*(*R*) at *R* = 1.0 nm is attributable to the reduced stabilization in the solvent-induced interactions for the transition state.

### Effect of temperature

**Figure 3:**
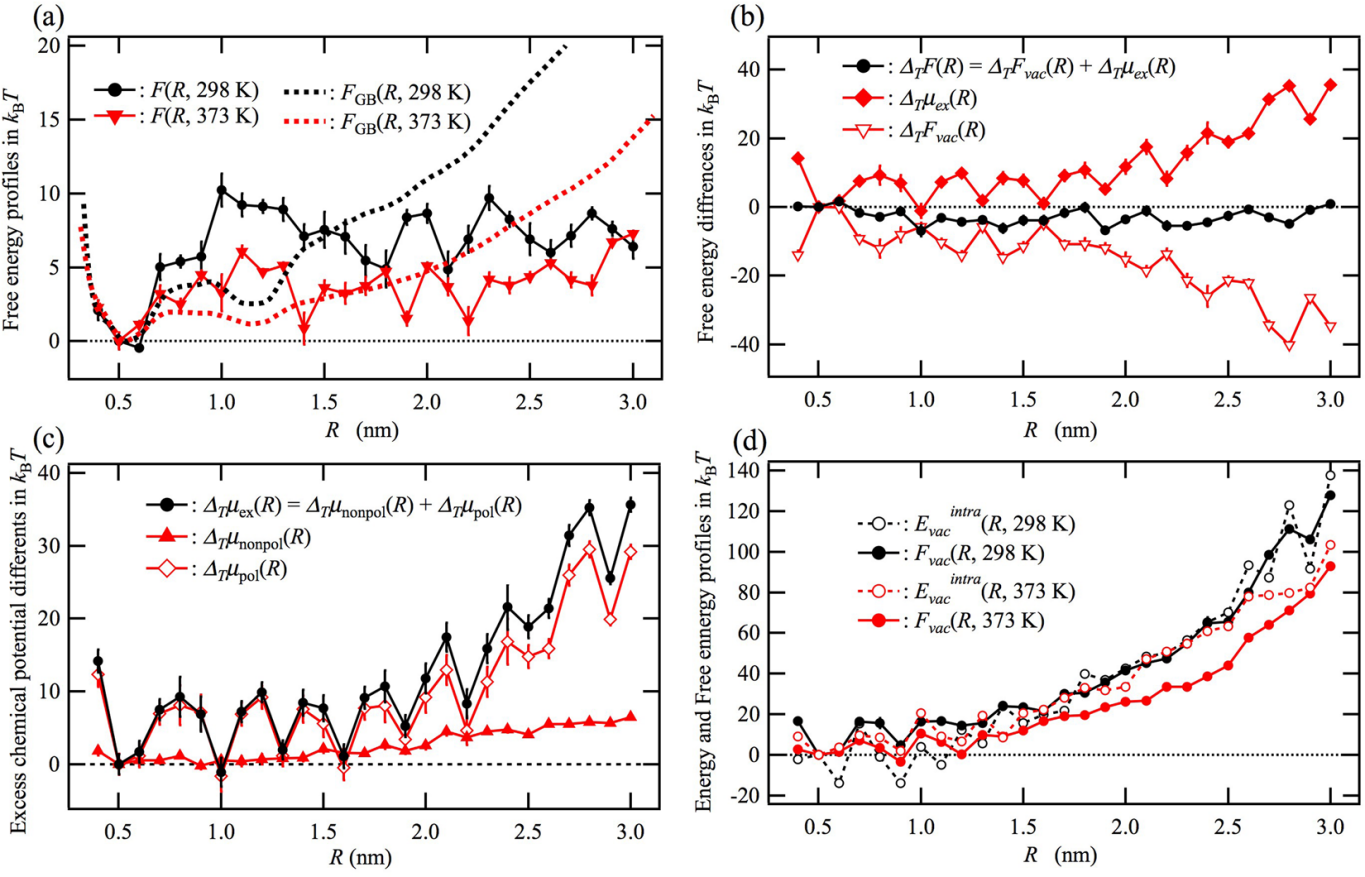
Comparison of free energy profiles for chignolin in water at 298 K and 373 K. (**a**) Free energy profile calculated according to the GB model, *F*_GB_(*R*), and that corrected by the RMDFT method, *F*(*R*). (**b**) Difference between the free energy profiles at 373 K and 298 K, $${{\rm{\Delta }}}_{T}F(R)=F(R,373\,{\rm{K}})/{k}_{B}T-F(R,298\,{\rm{K}})/{k}_{B}T$$ and the corresponding differences for the two components, $${{\rm{\Delta }}}_{T}{F}_{vac}(R)$$ and $${{\rm{\Delta }}}_{T}{\mu }_{ex}(R)$$, where $${{\rm{\Delta }}}_{T}F(R)={{\rm{\Delta }}}_{T}{F}_{vac}(R)+$$$${{\rm{\Delta }}}_{T}{\mu }_{ex}(R)$$. (**c**) Nonpolar part and polar part for $${{\rm{\Delta }}}_{T}{\mu }_{ex}(R)$$, $${{\rm{\Delta }}}_{T}{\mu }_{nonpol}(R)$$ and $${{\rm{\Delta }}}_{T}{\mu }_{pol}(R)$$, respectively, where $${{\rm{\Delta }}}_{T}{\mu }_{ex}(R)={{\rm{\Delta }}}_{T}{\mu }_{nonpol}(R)+{{\rm{\Delta }}}_{T}{\mu }_{pol}(R)$$. (**d**) Free energy profile in vacuum, *F*_*vac*_(*R*), and its energy part, $${E}_{vac}^{intra}(R)$$, at 298 K and 373 K. The difference between *F*_*vac*_(*R*) and $${E}_{vac}^{intra}(R)$$ corresponds to the entropic term of *F*_*vac*_(*R*), $$-\,T{S}_{vac}^{intra}(R)={F}_{vac}(R)-{E}_{vac}^{intra}(R)$$ (see Eq. 2).

The red solid curve in Fig. 3a shows the RMDFT free energy profile *F*(*R*) at 373 K. The relative stability of unfolded conformations compared with that of the native state is lower at 373 K than at 298 K. The free energy change from the folded to unfolded state, ${\rm{\Delta }}{F}_{u}$, was experimentally determined by Honda *et al*.^33^ to be 0.5 *k*_B_*T* at 298 K and −2.2 *k*_B_*T* at 373 K. Not only the RMDFT method but also the generalized-ensemble all-atom MD simulation by Okumura^25^ estimated the ${\rm{\Delta }}{F}_{u}$ higher than the experimental values at both temperatures. On the other hand, the relative stabilization of the unfolded state by heating, ${{\rm{\Delta }}}_{T}{F}_{u}={\rm{\Delta }}{F}_{u}(373\,{\rm{K}})/{k}_{{\rm{B}}}T-{\rm{\Delta }}{F}_{u}(298\,{\rm{K}})/{k}_{{\rm{B}}}T$, where the former and latter were obtained as −0.1 and 2.7, respectively, was, thus, estimated by RMDFT to be −2.8, if we assume the sum of the state probabilities at *R* = 0.5 and 0.6 nm as the probability of the folded state. This value is comparable with the experimental value, −2.7.

Figure 3b shows the difference in the free energy profile between 373 K and 298 K, $${{\rm{\Delta }}}_{T}F(R)=F(R,373\,{\rm{K}})/$$$${k}_{B}T-F(R,298\,{\rm{K}})/{k}_{B}T$$, and the corresponding differences for the two components, $${{\rm{\Delta }}}_{T}{F}_{vac}(R)$$ and $${{\rm{\Delta }}}_{T}{\mu }_{ex}(R)$$, where $${{\rm{\Delta }}}_{T}F(R)={{\rm{\Delta }}}_{T}{F}_{vac}(R)+{{\rm{\Delta }}}_{T}{\mu }_{ex}(R)$$. $${{\rm{\Delta }}}_{T}{\mu }_{ex}(R)$$ increases with increasing *R* mainly because of the increase in the electrostatic part of $${{\rm{\Delta }}}_{T}{\mu }_{ex}(R)$$, $${{\rm{\Delta }}}_{T}{\mu }_{pol}(R)$$, as shown in Fig. 3c. This result shows that the electrostatic part of the solvent-induced interaction significantly suppresses the high-temperature unfolding. In contrast, $${{\rm{\Delta }}}_{T}{F}_{vac}(R)$$ decreases with increasing *R*, as seen in Fig. 3b, indicating that the stabilization of the unfolded state at high temperature is attributable to the intramolecular free energy *F*_*vac*_(*R*). In Fig. 3d, we show a comparison between *F*_*vac*_(*R*) and the energy part of *F*_*vac*_(*R*), $${E}_{vac}^{intra}(R)$$, at 298 K and 373 K. $${E}_{vac}^{intra}(R)$$ is similar at 373 K and 298 K, whereas the intramolecular free energy *F*_*vac*_(*R*) for the unfolded conformations is lower at 373 K than at 298 K. We, therefore, conclude that the dominant factor in high-temperature unfolding is intramolecular conformation entropy, $$-\,T{S}_{vac}^{intra}(R)$$ (see Eq. 2), because the intramolecular-conformation-entropy-driven unfolding slightly overcomes the solvent-induced collapse.

### Effect of pressure

**Figure 4:**
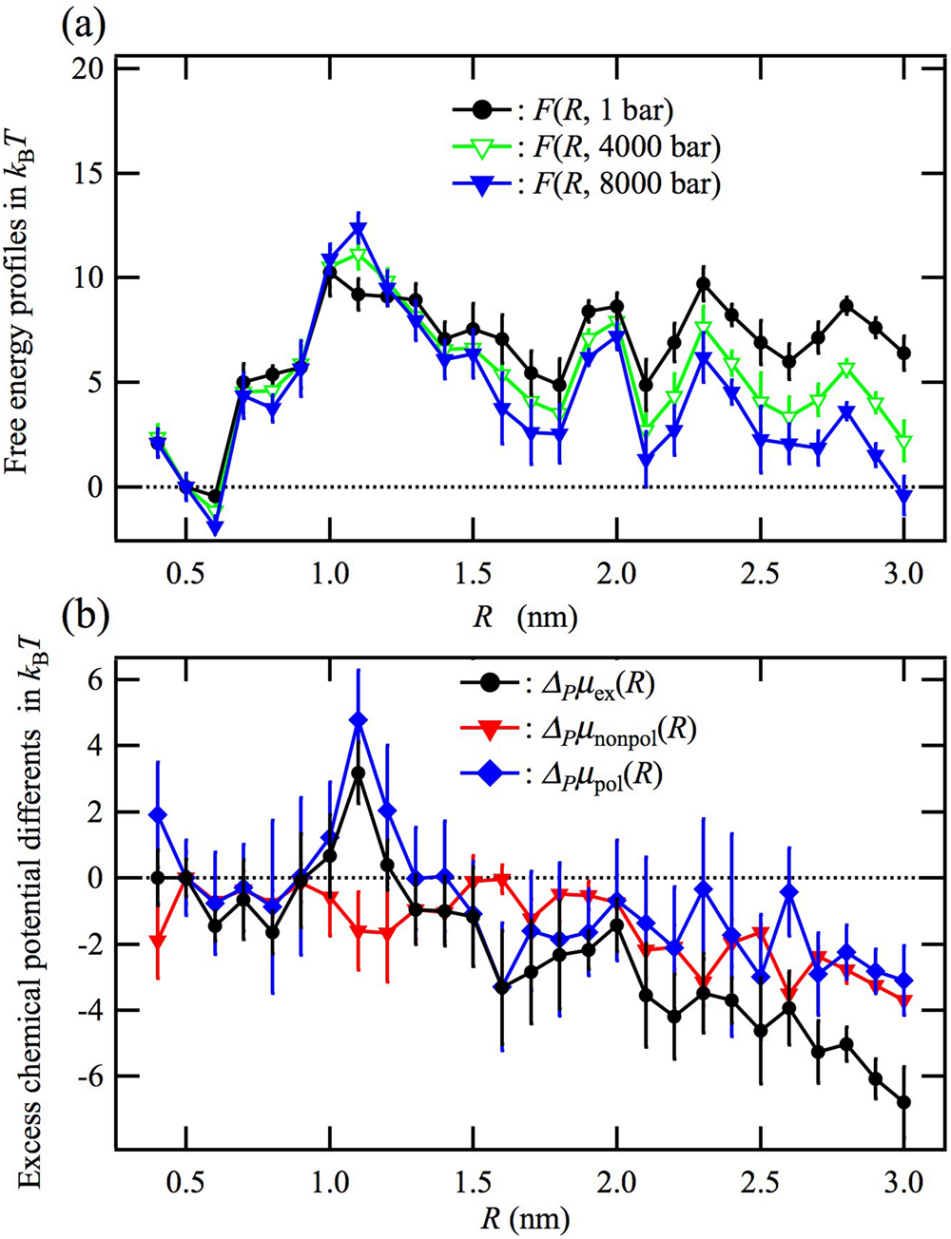
Effect of pressure on the free energy profile *F*(*R*). (**a**) Pressure dependence of the free energy profile at 298 K. (**b**) Difference in the free energy between 8000 bar and 1 bar, ${{\rm{\Delta }}}_{P}F(R)=F(R,8000\,{\rm{bar}})/{k}_{B}T-F(R,1\,{\rm{bar}})/{k}_{B}T$. Shown are the excess chemical potential difference, ${{\rm{\Delta }}}_{P}{\mu }_{ex}(R)$, the nonpolar part ${{\rm{\Delta }}}_{P}{\mu }_{nonpol}(R)$, and the electrostatic part, ${{\rm{\Delta }}}_{P}{\mu }_{pol}(R)={{\rm{\Delta }}}_{P}{\mu }_{ex}(R)-{{\rm{\Delta }}}_{P}{\mu }_{nonpol}(R)$, resulting from the independence of *F*_*vac*_(*R*) on pressure.

Figure 4a shows the pressure dependence of *F*(*R*) at 298 K. The unfolded state becomes more stable than the folded state with increasing pressure, although the barrier at *R* = 1.1 nm is somewhat raised by the pressurization. High-pressure unfolding of chignolin has previously been observed by FT-IR and FRET experiments^49^ and a similar behavior for *F*(*R*) has also been obtained by the generalized-ensemble all-atom MD simulations by Okumura^25^. The partial molar volume change from the folded to unfolded state, ${\rm{\Delta }}V={(\partial {\rm{\Delta }}{F}_{u}/\partial P)}_{T}$, which is a crucial thermodynamic quantity that characterizes the pressure-induced unfolding, was obtained by RMDFT as −5.3 cm3/mol in the same manner as the heat denaturation. This value is comparable with the all-atom MD simulation value obtained by Okumura, −5.6 cm^3^/mol^25^. Both the values are slightly larger than the experimental value, −8.8 cm^3^/mol^49^.

Figure 4b shows the difference in the free energy between 8000 bar and 1 bar, $${{\rm{\Delta }}}_{P}F(R)=F(R,8000\,{\rm{bar}})/$$$${k}_{B}T-F(R,1\,{\rm{bar}})/{k}_{B}T$$, which is identical to the difference in the excess chemical potential profile $${{\rm{\Delta }}}_{P}{\mu }_{ex}(R)$$ because *F*_*vac*_(*R*) is independent of pressure. The maximum of $${{\rm{\Delta }}}_{P}{\mu }_{ex}(R)$$ at a distance of *R* = 1.1 nm indicates that the conformations near the transition state have a larger excess partial molar volume than the other conformations including the native ones. This is because there would exist many narrow spaces that prevent water molecules from accessing the protein surface. It is remarkable that both the polar part of $${{\rm{\Delta }}}_{P}{\mu }_{ex}(R)$$, $${{\rm{\Delta }}}_{P}{\mu }_{pol}(R)$$, and the nonpolar part, $${{\rm{\Delta }}}_{P}{\mu }_{nonpol}(R)$$, are lower by 3~4 at a distance of *R* = 3.0 nm. The unfolded state, therefore, is stabilized at high pressure by both the nonpolar part^50^ and electrostatic part of the solvent-induced interaction. The decrease in the polar part, $${{\rm{\Delta }}}_{P}{\mu }_{pol}(R)$$, with increasing *R* under high pressure is consistent with the mechanism of high-pressure unfolding of proteins proposed by Chalikian and Macgregor^51^.

## Concluding Remarks

Kauzmann’s hydrophobic interaction hypothesis rests on the following assumption: either there is a precise compensation in the interaction energy between dehydration of polar groups and intramolecular hydrogen-bond formation or the effect of electrostatic interactions on protein stability is negligibly small. For chignolin, which is thought to possess both intramolecular hydrogen bonds and potential hydrophobic bonds in the native state^33^, it was shown that neither the nonpolar part nor the polar part of the solvent-induced interaction plays a predominant role in the collapse of chignolin. In fact, the solvent-induced interaction rather stabilizes the unfolded conformations mainly due to the electrostatic part of *μ*_*ex*_(*R*). Therefore, the dominant factor in the collapse of chignolin is the intramolecular interactions including van der Waals and electrostatic forces. Furthermore, the conformation of the native state is determined by the subtle balance between competitive factors, i.e., the intramolecular interactions and the solvent-induced interactions. It is remarkable that these results obtained for chignolin are consistent with the conclusions of previous studies on site-directed mutagenesis of globular proteins with interior hydrophobic cores^13,14^.

It has been demonstrated by calorimetry experiments that thermal unfolding of globular proteins is an endothermic and entropy-driven process, where *T*Δ*S*_*u*_ = Δ*H*_*u*_ > 0, if Δ*G*_*u*_ = 0, and where Δ*G*_*u*_, Δ*H*_*u*_, and Δ*S*_*u*_ are change in Gibbs energy, enthalpy, and entropy due to unfolding, respectively^52,53^. In contrast, low-temperature unfolding is, therefore, an exothermic and entropy-reduction process, while such an observation is limited due to freezing of protein solutions. Therefore, widely observed protein folding from high-temperature unfolded states is an exothermic process with a decrease in entropy. The intramolecular-force-driven collapse hypothesis extracted from chignolin is consistent with the exothermic behavior upon protein folding, even though chignolin has no hydrophobic interior cores. This hypothesis can also provide a proper explanation for the exothermic aggregation generally observed for heat-denatured proteins^53,54^. However, these arguments as well as the results obtained for chignolin do not necessary guarantee the validity of this hypothesis on the thermodynamic stability of general proteins, thus we need further investigations for several larger proteins to assess the general validity of this conclusion.

The thermodynamic stability mechanism presented on the basis of the competition between the intramolecular interactions and the solvent-induced interactions may underlay the remarkable successes of the protein tube-like model^55,56^ and the Go-like model^57,58^. Furthermore, the competition between these opposing factors would also provide a new insight into self-assembly of bio/soft materials; especially, the physical origin of their softness in aqueous solutions may be attributed to the solvent-induced interactions because amphiphilic polymers such as poly(N-isopropylacrylamide) that are solid in vacuum due to direct intramolecular and intermolecular interactions become soft and swollen when immersed in water.

In our previous study^32^, based on an effective energy defined as the sum of intramolecular interaction energy and solvation free energy of each conformation generated by MD simulations in water, we had obtained qualitatively equivalent results for the thermodynamic stability of chignolin, which, though, had significantly overestimated the stability of folded conformations [see the argument at the beginning of the Supplementary Information as well as ref.^32^]. Thus, in the present work, we developed the computational method on the direct free energy decomposition for flexible protein molecule and applied it to chignolin again. The direct free energy decomposition demonstrates that we can apply the effective energy analysis to determine the predominant factor in the thermodynamic stability of proteins instead of using the time-consuming direct free energy decomposition. Investigating whether the competition mechanism holds in the thermodynamic stability of proteins with hydrophobic interior cores as well, using these methods or more improved ones with respect to conformation sampling, is our future important project.

## Computational Details

### Molecular simulations

Isothermal MD simulations^59^ were performed using the Gromacs 5.0.7 suite^60^ with the generalized Born (GB)/surface area (SA) continuum solvent model^61^ and the Amber99SB force field^62^. The time step in the MD simulations was 1.0 fs. All intramolecular bonds were constrained using the LINCS algorithm^63^. Non-bonded interactions were not truncated. In all the GB MD simulations for the coordinate *R* except for umbrella sampling, the distance between the alpha carbon atoms at the C- and N-terminus was fixed by a constraint.

### Umbrella sampling

The free energy profile for the unfolding of chignolin in a continuum solvent described by the GB model, *F*_*GB*_(*R*), was calculated from a set of umbrella sampling MD simulations using the weighted histogram analysis method (WHAM)^64^. A harmonic potential with a force constant of 1875 kJ mol^−1^ nm^−2^ was applied for the distance between the alpha carbon atoms at the C-terminus and N-terminus. The histogram of the force on these atoms was obtained from a 50-ns simulation for every window. The spacing of the windows along the coordinate *R* was 0.0125 nm and the number of windows was 208. The standard deviation of *F*_*GB*_(*R*) was estimated by a bootstrap analysis^64^.

### RMDFT calculations

The hydration free energy of the RMDFT model, ${\rm{\Delta }}{G}_{hyd}^{{\rm{DFT}}}$, was calculated for 5000 conformations generated by a 50-ns GB MD production run after a 10-ns equilibration at each *R*. The details of the RMDFT model are given in Appendix C of the Supplementary Information and our previous study^32^. The standard deviation of ${\rm{\Delta }}{\mu }_{{\rm{DFT}}}^{{\rm{GB}}}(R)$ was evaluated by decomposing the 5000 conformations into five equal blocks.

### Excess chemical potential of the GB model, ${{\boldsymbol{\mu }}}_{{\bf{ex}}}^{{\bf{GB}}}({\boldsymbol{R}})$

Twelve intermediate states, *ε*_*r*_ = 40, 20, 10, 5, 4.2, 3.5, 2.9, 2.4, 2.0, 1.7, 1.4, and 1.2 were considered when calculating ${\mu }_{{ex}}^{{\rm{GB}}}(R)$. The GB MD simulations of the intermediate states were performed sequentially. The conformation obtained at the end of the simulation with one step higher *ε*_*r*_ value was used as the initial conformation of the simulation at one step lower *ε*_*r*_ value. An equilibration run was performed for 10 ns, and the data of 5000 conformations generated by the following 50-ns production run were used to calculate ${\rm{\Delta }}{\mu }_{{\varepsilon }_{j}}^{{\varepsilon }_{i}}(R)$ for every intermediate state. The standard deviation of ${\rm{\Delta }}{\mu }_{{\varepsilon }_{j}}^{{\varepsilon }_{i}}(R)$ was evaluated in the same manner as for ${\rm{\Delta }}{\mu }_{{\rm{DFT}}}^{{\rm{GB}}}(R)$.

### Nonpolar part of *μ*_ex_(*R*), μ_nonpol_(*R*)

${\rm{\Delta }}{\mu }_{80}^{1}(R)$ and ${\rm{\Delta }}{\mu }_{1}^{80}(R)$ were calculated from 5000 conformations generated by 50-ns production run without all partial charges on chignolin using the dielectric constants *ε*_*r*_ = 1 (in vacuum) and *ε*_*r*_ = 80 (with only the nonpolar SA term), respectively. *μ*_*nonpol*_(*R*) is given as the sum of $[{\rm{\Delta }}{\mu }_{80}^{1}(R)-{\rm{\Delta }}{\mu }_{1}^{80}(R)]/2$ and ${\rm{\Delta }}{\mu }_{{\rm{DFT}}}^{{\rm{GB}}}(R)$, as given by Eq. 7.

## Supplementary information

Supplementary Information
