# Supplementary material for: Theoretical analysis on thermodynamic stability of chignolin
Source: Sci Rep. 2019 Mar 26;9:5186. doi: 10.1038/s41598-019-41518-1 (PMC6435801; doi:10.1038/s41598-019-41518-1)
Supplement: Supplementary file 1 — Supplementary Information [file 41598_2019_41518_MOESM1_ESM.pdf]

Supplementary Information

Theoretical analysis on thermodynamic stability of chignolin

Tomonari Sumi and Kenichiro Koga

Research Institute for Interdisciplinary Science, Okayama University, 3-1-1 Tsushima-Naka, Kita-ku, Okayama 700-8530, Japan.
Department of Chemistry, Faculty of Science, Okayama University, 3-1-1 Tsushima-Naka, Kita-ku, Okayama 700-8530, Japan.

An example showing the importance of the conformation relaxation effect on $\mu_{ex}(R)$

As pointed out in the main text, $\mu_{ex}(R)$ is essentially different from a simple ensemble-average value of solvation free energy calculated using conformations generated under the coordinate R in solvent. This is because $\mu_{ex}(R)$ should include the effect of conformation relaxation during gradual annihilation of either the protein or all solvent molecules [1]. In fact, as recently reported for chignolin [2,3], a lack of the appropriate conformation ensemble average provided a large discrepancy between the effective energy, namely, the sum of the intramolecular energy and solvation free energy and experimental observation. If the ensemble average was simply evaluated using conformations generated in solvent instead of performing free energy perturbation (Eqs. 4, 5, and 7 in the main text), a difference in the effective energy between the native and unfolded conformations was obtained to be 38 k_BT at 298 K [3]. This value was several ten times larger than an experimentally determined free energy difference [4]. In addition to the effect of conformation entropy on $F_{vac}(R)$, the conformation relaxation effect in $\mu_{ex}(R)$ is important to reproduce the free energy difference between the native and unfolded states.

Results

2D free energy profile.

Figures S1 show the free energy profiles of chignolin on a 2D plane of the distance R between the alpha carbon atoms at the C-terminus and the N-terminus and the backbone dihedral angle ψ of Gly7, $F_2(R,\psi)/k_BT$. The native state is characterized by $R\sim$ 0.5 nm and $\psi\sim 0^\circ$ [5] and the misfolded state [5-8] is by $R\sim$ 0.6 nm and $\psi\sim -150^\circ$ [5]. The misfolded state is slightly more stable than the native state even at 298 K and 1 bar (Fig. S1a). This observation is consistent with the previous all-atom MD simulation study [5]. As temperature

increases at 1 bar, unfolded conformations become stable over the wide distance and angle (Fig. S1b). In contrast, as pressure increases at 298 K, some extended conformations at specific local dihedral angles become stable (Fig. S1c). In addition, the native state becomes unstable with increasing pressure, while the misfolded state is still stable at the high pressure. Similar temperature and pressure dependences have been observed in a generalized-ensemble MD simulation [9]. The good agreement with the previous MD simulation implies that the present method can provide reliable multidimensional free energy profiles.

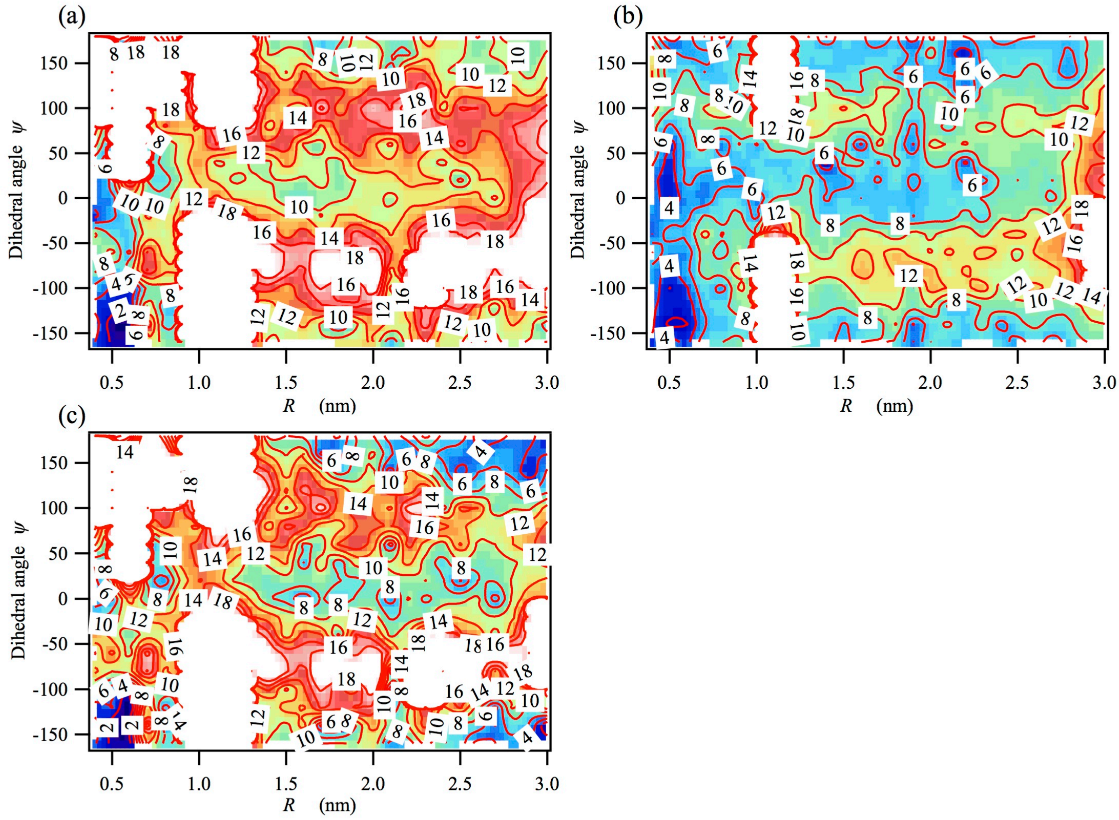

Figure S1. Two-dimensional free energy profiles of chignolin on a 2D plane of the end-to-end distance R and the backbone dihedral angle ψ of Gly7. (a) T= 298 K and P= 1 bar. (b) T= 298 K and P= 8000 bar. (c) T= 373 K and P= 1 bar. The native state is characterized by R~ 0.5 nm and ψ~ 0° [5] and the misfolded state [5-8] is by R~ 0.6 nm and ψ~ −150° [5].

Figures S2 show free energy profiles of chignolin on the 2D plane of the distance R and another backbone dihedral angle, ϕ of Gly7. The native state corresponds to R~ 0.5 nm and ϕ~ 100° [5], while the misfolded state [5-8] cannot be identified in this 2D free energy profile. We again observe that the unfolded conformations become stable over the wide distance R and angle ϕ by heating (Fig. S2b) while some specific extended states at large values of R become stable by pressurization (Fig. S2c).

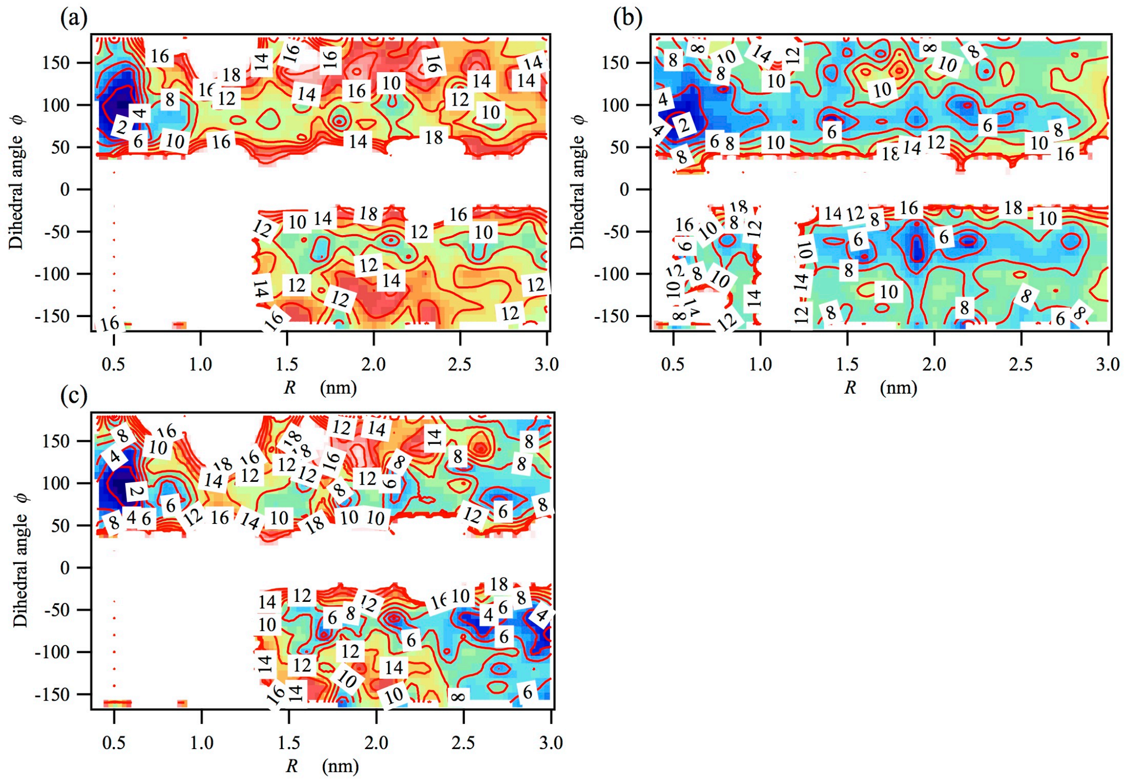

Figure S2. Free energy profiles of chignolin on the 2D plane of the end-to-end distance R and the backbone dihedral angle ϕ of Gly7. (a) T= 298 K and P= 1 bar. (b) T= 298 K and P= 8000 bar. (c) T= 373 K and P= 1 bar.

Influence of multiple states for $F_{vac}(R)$ and $\mu_{ex}^{GB}(R)$.

In order to calculate the excess chemical potential of the GB model, $\mu_{ex}^{\mathrm{GB}}(R)$, we performed GB MD simulations sequentially at ε_r= 80, 40, 20, 10, 5, 4.2, 3.5, 2.9, 2.4, 2.0, 1.7, 1.4, 1.2 and 1.0 for each R. The final conformation of the higher ε_r was used as the initial conformation of the lower ε_r. It is possible that there are multiple states for a given R value in vacuum and the conformational transitions between those states hardly occur, thus the difference in the conformations obtained in vacuum could provide an influence on $\mu_{ex}^{\mathrm{GB}}(R)$ and $F_{vac}(R)$. We performed the other set of GB MD simulations to calculate $\mu_{ex}^{\mathrm{GB}}(R)$ independently. As shown in Figure S3a, the two sets of simulations yield almost the same results. We also show the corresponding intramolecular energy profiles in vacuum, $E_{vac}^{intra}(R)$, in Fig. S3b. Again, we confirm no significant difference between these results.

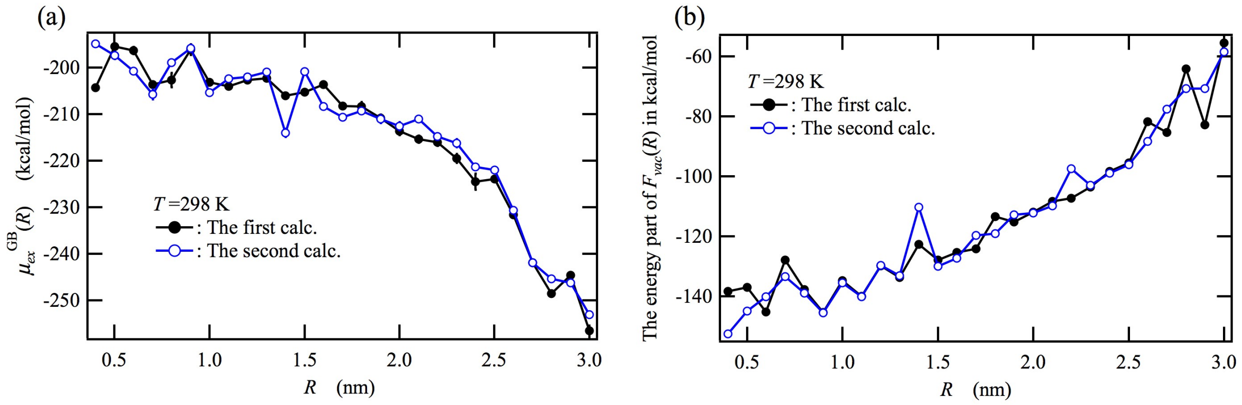

Figure S3. (a) $\mu_{ex}^{\mathrm{GB}}(R)$ and (b) the energy part of $F_{vac}(R)$, $E_{vac}^{intra}(R)$, calculated from two independent sets of GB MD simulations.

Appendix A. Relationship between the solvation free energy and a partition function of a protein in solvent.

A partition function of a protein in a solvent, Q_p, is given by [10]

$$Q_p = \Lambda_p^{-3P} \int d\mathbf{r}^P \exp(-\beta E_{st}[\{\mathbf{r}^P\}])\Xi_s\left[U_\lambda^{PR}(\mathbf{r}^P)\right]. \tag{A1}$$

Here, P is the number of atoms in the protein, Λ_p is the thermal wave length, $\beta \equiv 1/k_B T$ where k_B is the Boltzmann's constant, $E_{st}[\{\mathbf{r}^P\}]$ is the intramolecular energy of the protein, and $\Xi_s\left[U_\lambda^{PR}(\mathbf{r}^P)\right]$ is the grand canonical partition function of the solvent under an external field, $U_\lambda^{PR}(\mathbf{r}^P)$, which is caused by the protein-solvent interactions. This equation can be recast as

$$Q_p = \Lambda_p^{-3P} \int d\mathbf{r}^P \exp(-\beta E_{st}[\{\mathbf{r}^P\}])\exp\left(-\beta\Omega_s\left[U_\lambda^{PR}(\mathbf{r}^P)\right]\right) \tag{A2}$$

with

$$\Omega_s\left[U_\lambda^{PR}(\mathbf{r}^P)\right] = -k_B T \ln \Xi_s\left[U_\lambda^{PR}(\mathbf{r}^P)\right]. \tag{A3}$$

The solvation free energy of the protein in a given conformation $\mathbf{r}^P$ is given by

$$\Delta G_{solv}[\{\mathbf{r}^P\}] = \Omega_s\left[U_\lambda^{PR}(\mathbf{r}^P)\right] - \Omega_s[0]. \tag{A4}$$

The partition function Q_p can be expressed by using $\Delta G_{solv}[\{\mathbf{r}^P\}]$,

$$\begin{aligned} Q_p &= \Lambda_p^{-3P}\exp(-\beta\Omega_s[0]) \int d\mathbf{r}^P \exp(-\beta E_{st}[\{\mathbf{r}^P\}])\exp\left\{-\beta\left(\Omega_s\left[U_\lambda^{PR}(\mathbf{r}^P)\right] - \Omega_s[0]\right)\right\} \\ &= \Lambda_p^{-3P}\Xi_s[0] \int d\mathbf{r}^P \exp(-\beta E_{st}[\{\mathbf{r}^P\}])\exp(-\beta\Delta G_{solv}[\{\mathbf{r}^P\}]) \\ &= Q_p[0]\langle\exp(-\beta\Delta G_{solv}[\{\mathbf{r}^P\}])\rangle_{\mathbf{r}^P}, \end{aligned} \tag{A5}$$

where $\langle \quad \rangle_{\mathbf{r}^P}$ denotes an ensemble average with respect to the conformation of the protein in vacuum and

$$Q_p[0] \equiv \Lambda_p^{-3P} \int d\mathbf{r}^P \exp(-\beta E_{st}[\{\mathbf{r}^P\}])\exp\{-\beta(\Omega_s[0])\}. \tag{A6}$$

Equation A6 is a partition function for the pure solvent plus the protein in vacuum. The excess chemical potential of the protein is defined by the free energy difference between the pure solvent plus the protein in vacuum and the protein immersed in the solvent,

$$\mu_{ex} = -k_B T \ln Q_p + k_B T \ln Q_p[0] = -k_B T \ln\langle\exp(-\beta\Delta G_{solv}[\{\mathbf{r}^P\}])\rangle_{\mathbf{r}^P}. \tag{A7}$$

This equation can be also regarded as a one-step free energy perturbation method to determine the free energy difference, where the vacuum phase is the initial state and the solution phase is the final state. In general, the distribution of the protein conformation with lower intramolecular energy of the protein in vacuum has no enough overlap with the conformation distribution for lower solvation free energy of the protein in the solvent. We, thus, apply the free energy perturbation method given by Eqs. 4 and 5 to the calculation of the ensemble average of Eq. A7.

Appendix B. Free energy profile in a two-dimensional (2D) plane

We choose the distance between the alpha carbon atoms at the C-terminus side and at the N-terminus side for chignolin, as the primary coordinate, R. The one-dimensional probability distribution $P_1(R)$ is defined as

$$F(R) = -k_B T \ln P_1(R). \quad \text{(B1)}$$

We choose the backbone dihedral angle ψ of Gry7 as the secondary coordinate. The relation between the 1D and 2D distribution probabilities is given by

$$P_1(R) = \int d\psi\, P_2(R,\psi), \quad \text{(B2)}$$

and the 2D free energy is expressed as

$$F_2(R,\psi) = -k_B T \ln P_2(R,\psi). \quad \text{(B3)}$$

We introduce a normalized distribution function as

$$\overline{P_2}(R,\psi) = P_2(R,\psi) / \int d\psi\, P_2(R,\psi). \quad \text{(B4)}$$

Using Eqs. (B3) and (B4), we obtain

$$F_2(R,\psi) = -k_B T \ln\left(\overline{P_2}(R,\psi) \int d\psi\, P_2(R,\psi)\right)$$
$$= -k_B T \ln\left(\int d\psi\, P_2(R,\psi)\right) - k_B T \ln \overline{P_2}(R,\psi). \quad \text{(B5)}$$

Substituting Eq. (B2) into Eq. (B5) and using Eq. (B1), we finally obtain

$$F_2(R,\psi) = F(R) - k_B T \ln \overline{P_2}(R,\psi). \quad \text{(B6)}$$

Appendix C. Computational method of $\Delta G_{hyd}^{\mathrm{DFT}}$

In this study, we applied the reference-modified density functional theory (RMDFT) [11-13] to calculate the solvation free energy of chignolin in water for conformations generated by the MD simulations of the generalized Born (GB)/surface area (SA) continuum solvent model [14]. We employed the effective-density approximation (EDA) [15] to prepare the excess intrinsic free energy functional for the reference hard-sphere (HS) system, $F_{HS}^{ex}[n]$. The reliability of the RMDFT has been assessed by comparing with experiments [2,11]. The RMDFT hydration free energy is given by

$$\Delta G_{hyd}^{\mathrm{DFT}} = -\frac{1}{\beta}\sum_{a=1}^{P}\int d\mathbf{r}_1^a \left[n_a\left(\mathbf{r}_1^a\middle|\{U_\lambda^{PR}\}\right) - n_0\right] + \int d\mathbf{r}_1^{\mathrm{O}} \left[n_{\mathrm{O}}\left(\mathbf{r}_1^{\mathrm{O}}\middle|\{U_\lambda^{PR}\}\right) f_{HS}\left(n_{\mathrm{O}}^{eff}\left(\mathbf{r}_1^{\mathrm{O}}\middle|n_{\mathrm{O}}\right)\right) - n_0 f_{HS}(n_0)\right]$$

$$- \int d\mathbf{r}_1^{\mathrm{O}} \left[n_{\mathrm{O}}\left(\mathbf{r}_1^{\mathrm{O}}\middle|\{U_\lambda^{PR}\}\right)\left\{f_{HS}\left(n_{\mathrm{O}}^{eff}\left(\mathbf{r}_1^{\mathrm{O}}\middle|n_{\mathrm{O}}\right)\right)\right.\right.$$

$$\left.+ \int d\mathbf{r}_2^{\mathrm{O}}\, W_{\mathrm{OO}}\left(\left|\mathbf{r}_1^{\mathrm{O}} - \mathbf{r}_2^{\mathrm{O}}\right|\right) n_{\mathrm{O}}\left(\mathbf{r}_2^{\mathrm{O}}\middle|\{U_\lambda^{PR}\}\right) f'_{HS}\left(n_{\mathrm{O}}^{eff}\left(\mathbf{r}_2^{\mathrm{O}}\middle|n_{\mathrm{O}}\right)\right)\right\}$$

$$\left. - n_0\left\{f_{HS}(n_0) + n_0 f'_{HS}(n_0)\int d\mathbf{r}_2^{\mathrm{O}}\, W_{\mathrm{OO}}\left(\left|\mathbf{r}_1^{\mathrm{O}} - \mathbf{r}_2^{\mathrm{O}}\right|\right)\right\}\right]$$

$$+ \frac{n_0}{\beta}\sum_{a=1}^{P}\sum_{b=1}^{P}\int d\mathbf{r}_1^a d\mathbf{r}_1^b\, C_{ab}^{ex}\left(\left|\mathbf{r}_1^a - \mathbf{r}_2^b\right|\right)\left[n_b\left(\mathbf{r}_1^b\middle|\{U_\lambda^{PR}\}\right) - n_0\right]$$

$$+ \frac{1}{2\beta}\sum_{a=1}^{P}\sum_{b=1}^{P}\int d\mathbf{r}_1^a d\mathbf{r}_1^b\, C_{ab}^{ex}\left(\left|\mathbf{r}_1^a - \mathbf{r}_2^b\right|\right)\left[n_a\left(\mathbf{r}_1^a\middle|\{U_\lambda^{PR}\}\right) - n_0\right]\left[n_b\left(\mathbf{r}_1^b\middle|\{U_\lambda^{PR}\}\right) - n_0\right]$$

(C1)

Here, $f'_{HS}(n)$ is the first derivative of $f_{HS}(n)$, which is the excess free energy of the HS system per particle. A highly accurate expression for $f_{HS}(n)$, obtained from the Carnahan–Starling (CS) equation of state,[16,17] is available:

$$f_{HS}(n) \equiv \frac{A_{HS}^{ex}}{N} = \frac{1}{\beta}\frac{\eta(4-3\eta)}{(1-\eta)^2}, \tag{C2}$$

$$= \pi n_0 (d_{HS})^3/6, \tag{C3}$$

where d_{HS} is the diameter of the reference HS fluid. $n_{\mathrm{O}}^{eff}\left(\mathbf{r}_1^{\mathrm{O}}\middle|n_{\mathrm{O}}\right)$ in Eq. (C1) is defined by the EDA excess intrinsic free energy functional for the reference HS system:

$$F_{HS}^{ex}[n_O] = \int d\mathbf{r}_1^{\mathrm{O}}\, n_{\mathrm{O}}\left(\mathbf{r}_1^{\mathrm{O}}\middle|\{U_\lambda^{PR}\}\right) f_{HS}\left(n_{\mathrm{O}}^{eff}\left(\mathbf{r}_1^{\mathrm{O}}\middle|n_{\mathrm{O}}\right)\right), \tag{C4}$$

where $n_{\mathrm{O}}^{eff}\left(\mathbf{r}_1^{\mathrm{O}}\middle|n_{\mathrm{O}}\right)$ is the effective density, which is assumed to be a functional of $n_{\mathrm{O}}\left(\mathbf{r}\middle|\{U_\lambda^{PR}\}\right)$. $n_{\mathrm{O}}^{eff}(\mathbf{r}|n_{\mathrm{O}})$ is approximated by the first-order density functional Taylor series expansion:

$$n_{\mathrm{O}}^{eff}(\mathbf{r}|n_{\mathrm{O}}) = n_0 + \int d\mathbf{r}_1^{\mathrm{O}}\, W_{\mathrm{OO}}\left(\left|\mathbf{r} - \mathbf{r}_1^{\mathrm{O}}\right|\right)\left[n_{\mathrm{O}}\left(\mathbf{r}_1^{\mathrm{O}}\middle|\{U_\lambda^{PR}\}\right) - n_0\right]. \tag{C5}$$

The expansion coefficient $W_{\mathrm{OO}}(r)$ also appears in Eq. (C1) and is related to the second-order direct correlation function $C_{OO}^{HS}(r)$ for the reference HS fluid via

$$\widehat{W}_{\mathrm{OO}}(k) = \left[-2\beta f'_{HS}(n_0) + \sqrt{\left(2\beta f'_{HS}(n_0)\right)^2 - 4n_0\beta f''_{HS}(n_0)\hat{C}_{OO}^{HS}(k)}\right]/2n_0\beta f''_{HS}(n_0), \tag{C6}$$

where $\widehat{W}_{\mathrm{OO}}(k)$ and $\hat{C}_{OO}^{HS}(k)$ are the Fourier transforms of $W_{OO}(r)$ and $C_{OO}^{HS}(r)$, respectively, and $f''_{HS}(n)$ is the second derivative of $f_{HS}(n)$. The density functional differentiation of $F_{HS}^{ex}[n_O]$ appearing in Eq. (C1) is obtained from Eq. (C4) as follows:

$$\frac{\delta F_{HS}^{ex}[n_O]}{\delta n_O\left(\mathbf{r}\middle|\{U_\lambda^{PR}\}\right)} = f_{HS}\left(n_{\mathrm{O}}^{eff}(\mathbf{r}|n_{\mathrm{O}})\right) + \int d\mathbf{r}_1^{\mathrm{O}}\, W_{\mathrm{OO}}\left(\left|\mathbf{r} - \mathbf{r}_1^{\mathrm{O}}\right|\right) n_{\mathrm{O}}\left(\mathbf{r}_1^{\mathrm{O}}\middle|\{U_\lambda^{PR}\}\right) f'_{HS}\left(n_{\mathrm{O}}^{eff}(\mathbf{r}|n_{\mathrm{O}})\right). \tag{C7}$$

In this study, we calculated the site-site direct correlation functions $\{C_{\alpha\beta}(r)\}$ for bulk water and the

site-density distribution functions of water around a solute molecule $\{n_\alpha(\mathbf{r}|\{U_\lambda^{PR}\})\}$ using the 1D-RISM-KH and 3D-RISM-KH integral equations [18,19], respectively. Before we calculate $\Delta G_{hyd}^{\mathrm{DFT}}$ from Eq. (C1) using the sets of $\{C_{\alpha\beta}(r)\}$ and $\{n_\alpha(\mathbf{r}|\{U_\lambda^{PR}\})\}$, it is necessary to determine $W_{\mathrm{OO}}(r)$ or $C_{OO}^{HS}(r)$ by solving the Ornstein-Zernike (OZ) integral equation,[16] in which the following EDA equation combined with the Percus' relation [16,20] is used as the closure:

$$H^{HS}(r) = n(\mathbf{r}|U_{PR}^{HS})/n_0 - 1, \qquad \text{(C8)}$$

$$n(r|U_{PR}^{HS}) = n_0 exp(-\beta U^{IG}(r)), \qquad \text{(C9)}$$

$$\begin{aligned} U^{IG}(r) &= U_{PR}^{HS}(r) + \frac{\delta F_{HS}^{ex}[n]}{\delta n(\mathbf{r}|\{U_{PR}^{HS}\})} - \mu^{exHS} \\ &= U_{PR}^{HS}(r) + f_{HS}\left(n_{\mathrm{O}}^{eff}(r|n_{\mathrm{O}})\right) - f_{HS}(n_0) \\ &= \int d\mathbf{r}_1\, W(|\mathbf{r}-\mathbf{r}_1|)\left[n(\mathbf{r}_1|U_{PR}^{HS})f_{HS}'\left(n_{\mathrm{O}}^{eff}(\mathbf{r}_1|n)\right) - n_0 f_{HS}'(n_0)\right], \end{aligned} \qquad \text{(C10)}$$

$$\hat{H}^{HS}(k) = \hat{C}^{HS}(k) + n_0\hat{C}^{HS}(k)\hat{H}^{HS}(k). \qquad \text{(C11)}$$

Here, $U_{PR}^{HS}(r)$ by the Percus' relation in Eq. (C8) is equal to the interaction between HS particles of the reference system.

Appendix D. Calculation details of $\Delta G_{hyd}^{\mathrm{DFT}}$

To calculate the site-density distribution functions $n_\alpha(\mathbf{r}|\{U_\lambda^{PR}\})$ in Eq. (C1), we applied the three-dimensional reference-interaction-site-model (3D-RISM) integral equation theory [18,19]. We employed the partially linearized HNC (PLHNC) equation [18], called the Kovalenko–Hirata (KH) equation [19], as the closure relations for both the 1D-RISM equation for bulk water and the 3D-RISM equation for solute–solvent systems. As for the model water in these RISM calculations, we used the TIP3P model with an additional LJ parameter for the hydrogen sites (d_H = 0.4 Å and $\varepsilon_H = 0.046\ Kcal/mol$) [21,22]. The solute–solvent cross parameters were deduced from the Lorentz–Berthelot mixing rules, $d_{ij} = (d_{ii} + d_{jj})/2$ and $\varepsilon_{ij} = \sqrt{\varepsilon_{ii}\varepsilon_{jj}}$, commonly introduced as the solute–solvent combination rule in the RISM calculations. The 3D-RISM integral equations were solved in a cubic cell with a size of 80 $Å^3$ using a grid of 256^3 points by utilizing graphics processing unit (GPU) [23]. In the same manner as our previous work [13], we used 0.00125 Å and 32768 as the grid spacing and the number of grids, respectively, to solve the 1D-RISM equation for bulk water and the EDA equation for the reference HS system. The number densities of water and the optimal HS diameters used for the thermodynamic states at 298 K and 1 bar, at 373 K and 1 bar, at 298 K and 4000 bar, and at 298 K and 8000 bar have been provided by our previous work [13].

References

(1) Yu, H. A.; Karplus, M. A Thermodynamic Analysis of Solvation. *J Chem Phys* **1988**, *89* (4), 2366–2379.

(2) Sumi, T.; Maruyama, Y.; Mitsutake, A.; Mochizuki, K.; Koga, K. Application of

Reference-Modified Density Functional Theory: Temperature and Pressure Dependences of Solvation Free Energy. *J. Comput. Chem.* **2018**, *39* (4), 202–217.

(3) Maruyama, Y.; Mitsutake, A. Analysis of Structural Stability of Chignolin. *J Phys Chem B* **2018**, *122* (14), 3801–3814.

(4) Honda, S.; Yamasaki, K.; Sawada, Y.; Morii, H. 10 Residue Folded Peptide Designed by Segment Statistics. *Structure* **2004**, *12* (8), 1507–1518.

(5) Kührová, P.; De Simone, A.; Otyepka, M.; Best, R. B. Force-Field Dependence of Chignolin Folding and Misfolding: Comparison with Experiment and Redesign. *Biophys. J.* **2012**, *102* (8), 1897–1906.

(6) Satoh, D.; Shimizu, K.; Nakamura, S.; Terada, T. Folding Free-Energy Landscape of a 10-Residue Mini-Protein, Chignolin. *FEBS Lett* **2006**, *580* (14), 3422–3426.

(7) Harada, R.; Kitao, A. Exploring the Folding Free Energy Landscape of a Β-Hairpin Miniprotein, Chignolin, Using Multiscale Free Energy Landscape Calculation Method. *J Phys Chem B* **2011**, *115* (27), 8806–8812.

(8) Mitsutake, A.; Takano, H. Relaxation Mode Analysis and Markov State Relaxation Mode Analysis for Chignolin in Aqueous Solution Near a Transition Temperature. *The Journal of Chemical Physics* **2015**, *143* (12), 124111–124111.

(9) Okumura, H. Temperature and Pressure Denaturation of Chignolin: Folding and Unfolding Simulation by Multibaric-Multithermal Molecular Dynamics Method. *Proteins* **2012**, *80* (10), 2397–2416.

(10) Sumi, T.; Sekino, H. A Cooperative Phenomenon Between Polymer Chain and Supercritical Solvent: Remarkable Expansions of Solvophobic and Solvophilic Polymers. *The Journal of Chemical Physics* **2005**, *122* (1), 4910.

(11) Sumi, T.; Mitsutake, A.; Maruyama, Y. A Solvation-Free-Energy Functional: a Reference-Modified Density Functional Formulation. *J. Comput. Chem.* **2015**, *36* (18), 1359–1369.

(12) Sumi, T.; Mitsutake, A.; Maruyama, Y. Erratum: "a Solvation-Free-Energy Functional: a Reference-Modified Density Functional Formulation" [J. Comput. Chem. 2015, 36, 1359–1369]. *J. Comput. Chem.* **2015**, *36* (26), 2009–2011.

(13) Sumi, T.; Maruyama, Y.; Mitsutake, A.; Mochizuki, K.; Koga, K. Application of Reference-Modified Density Functional Theory: Temperature and Pressure Dependences of Solvation Free Energy. *J. Comput. Chem.* **2018**, *39* (4), 202–217.

(14) Qiu, D.; Shenkin, P. S.; Hollinger, F. P.; Still, W. C. The GB/SA Continuum Model for Solvation. a Fast Analytical Method for the Calculation of Approximate Born Radii. *The Journal of Physical Chemistry A* **1997**, *101*, 3005–3014.

(15) Sumi, T.; Sekino, H. A Self-Consistent Density-Functional Approach for Homogeneous and Inhomogeneous Classical Fluids. *J. Phys. Soc. Jpn.* **2008**, *77* (3), 034605.

(16) Hansen, J.-P.; McDonald, I. R. *Theory of Simple Liquids; 2nd Ed.*; Academic Press: London, 1986.

(17) Carnahan, N. F.; Starling, K. E. Equation of State for Nonattracting Rigid Spheres. *The Journal of Chemical Physics* **1969**, *51*, 635–636.

(18) Kovalenko, A.; Hirata, F. Self-Consistent Description of a Metal-Water Interface by the Kohn-Sham Density Functional Theory and the Three-Dimensional Reference Interaction Site Model. *The Journal of Chemical Physics* **1999**, *110* (2), 10095–10112.

(19) *Molecular Theory of Solvation*; Hirata, F., Ed.; Kluwer Academic Publishers: Dordrecht, 2003; Vol. 24.

(20) Percus, J. K. Approximation Methods in Classical Statistical Mechanics. *Phys. Rev. Lett.* **1962**, *8* (1), 462–463.

(21) Jorgensen, W. L.; Chandrasekhar, J.; Madura, J. D.; Impey, R. W.; Klein, M. L. Comparison of Simple Potential Functions for Simulating Liquid Water. *J Chem Phys* **1983**, *79* (2), 926.

(22) Pettitt, B. M.; Rossky, P. J. Integral Equation Predictions of Liquid State Structure for Waterlike Intermolecular Potentials. *J Chem Phys* **1982**, *77* (3), 1451–1457.

(23) Maruyama, Y.; Hirata, F. Modified Anderson Method for Accelerating 3D-RISM Calculations Using Graphics Processing Unit. *J. Chem. Theory Comput.* **2012**, *8* (9), 3015–3021.
